# Supplementary material for: Combining ablative radiotherapy and anti CD47 monoclonal antibody improves infiltration of immune cells in tumor microenvironments
Source: PLoS One. 2022 Aug 26;17(8):e0273547. doi: 10.1371/journal.pone.0273547 (PMC9417014; doi:10.1371/journal.pone.0273547)
Supplement: S1 Checklist — (DOC) [file pone.0273547.s001.doc]

# *PLOS ONE Humane Endpoints Checklist*

*PLOS ONE manuscript number:* **PONE-D-22-01036**

**Complete the following if your study design includes death of a regulated animal as a likely outcome or planned experimental endpoint. Please also include all information in the Methods section of your manuscript.**

**ITEM 1.** **Describe whether humane endpoints* were used for all animals involved in the study.**

|  | **Recommendation** | **Section/Paragraph** |
| --- | --- | --- |
| **If humane endpoints* were used, report the following:** | | |
| **1** | **The specific criteria used to determine when animals should be euthanized** | - tumor growth greater than 1500 mm^3^  - bodyweight decreases by more than 15% of the initial weight  - health decline  - Visible symptoms of sickness***.*** |
| **2** | **Once animals reached endpoint criteria, the amount of time elapsed before euthanasia** | **Mice were examined regularly and sacrificed in less than 3 days until the endpoint.** |
| **3** | **Whether any animals died before meeting criteria for euthanasia** | **No** |
| **If humane endpoints* were not used, report the following:** | | |
| **1** | **A scientific and ethical justification for the study design, including the reasons why humane endpoints could not be used, and discussion of alternatives that were considered but could not be used** |  |
| **2** | **Whether the institutional animal ethics committee specifically reviewed and approved the anticipated mortality in the study design** | Institutional Ethical Committee and Research Advisory Committee of Mashhad University of Medical Sciences  Ethic Number code: IR.MUMS.fm.REC.1396.495 |

**ITEM 2.** **Include the following details of the study design and outcomes.**

|  | **Recommendation** | **Section/Paragraph** |
| --- | --- | --- |
| **1** | **The duration of the experiment** | ***90 days*** |
| **2** | **The numbers of animals used, euthanized, and found dead (if any); the cause of death for all animals** | ***40 mice were used for study***   - ***24 mice were used for in vivo study*** - ***12 mice were used for in vitro study*** - ***1 mice died during radiation therapy*** - ***4 mice were lack of tumors and were sacrificed*** |
| **3** | **How frequently animal health and behavior were monitored** | **Three times a week *mice health were surveyed.*** |
| **4** | **All animal welfare considerations taken, including efforts to minimize suffering and distress, use of analgesics or anaesthetics, or special housing conditions** | **Mice were kept in the animal room for two weeks to adapt to the environment. Tumor induction under anesthetized conditions was then performed and four groups were randomly selected.**  Mice radiation also was performed under anesthetized conditions. Mice were controlled four hours after radiation and pain symptoms were evaluated. If symptoms of pain such as anorexia, paleness, weight loss, and lumbar curvature were observed the pains were relieved.  After observing the endpoint criteria, mice were anesthetized with isoflurane and sacrificed ten minutes later. |
| **5** | **Any special training in animal care or handling provided for research staff** | **Special training for working with animals such as proper respiratory and injection anesthesia, how to fix the mice in the tube and control radiation conditions, painless death, and victimization were taught to the project staff.** |
|  |  |  |

***Definition of a humane endpoint**

A humane endpoint is an experimental endpoint at which animals are euthanized when they display early markers associated with death or poor prognosis of quality of life, or specific signs of severe suffering or distress. Humane endpoints are used as an alternative to allowing such conditions to continue or progress to death following the experimental intervention (“death as an endpoint”), or only euthanizing animals at the end of an experiment. Before a study begins, researchers define the practical observations or measurements that will be used during the study to recognize a humane endpoint, based on anticipated clinical, physiological, and behavioral signs. These may include, for instance, body temperature or weight changes, tumor size or appearance, abnormal behaviors, pathological changes, ruffled fur, reduced mobility, body posture, or expression of specific body fluid markers. Please see the NC3Rs guidelines for more information.

**ARRIVE Guidelines**

*PLOS ONE* encourages authors to follow the [Animal Research: Reporting of In Vivo Experiments (ARRIVE) guidelines](http://www.nc3rs.org.uk/arrive-guidelines) for all submissions describing laboratory-based animal research and to upload a completed [ARRIVE Guidelines Checklist](http://www.nc3rs.org.uk/sites/default/files/documents/Guidelines/NC3Rs ARRIVE Guidelines Checklist (fillable).pdf) to be published as supporting information.
