# Supplementary material for: Combining ablative radiotherapy and anti CD47 monoclonal antibody improves infiltration of immune cells in tumor microenvironments
Source: PLoS One. 2022 Aug 26;17(8):e0273547. doi: 10.1371/journal.pone.0273547 (PMC9417014; doi:10.1371/journal.pone.0273547)
Supplement: Attachment — Submitted filename: Response to reviewer.docx [file pone.0273547.s002.docx]

Jul 26, 2022

Dr. Norikatsu Miyoshi
Academic Editor

PLOS ONE

Thank you very much for your letter regarding our manuscript (PONE-D-22-01036) entitled" **Combining Ablative Radiotherapy and Anti CD47 Monoclonal Antibody Improves Infiltration of Immune Cells in Tumor Microenvironments**". The reviewers’ comments were very helpful, and we made revisions to the manuscript according to their criticisms.

I think described point together with the other points that we have addressed made the manuscript much improved. Please find attached a detailed reply to the referees’ comments and a description of other changes to the manuscript.

**Reviewer #4**: The article is in line with the current hot topics of radiotherapy activating immunity. Using radiotherapy and single-targeted immunotherapy in combination, it is very inspiring to draw the conclusion that this combination regimen is superior to radiotherapy or immunotherapy alone. But there are still some details to discuss in this article:

1. The author's intention of using high-dose irradiation alone as part of a combined regimen is based on previous published results, from high-dose alone, or hypo fractionation, or multiple fractions. The one with the most enrichment of immune cells in the tumor microenvironment after radiation has been selected. But not all radiation regimens have been considered, such as low-dose 1-2 Gy radiation which can induce more immune cells, or flash radiation which has been reported to have effect of activating abscopal effect.

Response: Thank you for your close attention. This type of radiotherapy will be investigated in a study that is currently being conducted by our group.

2. The recruitment of CD8^+^ cells increases after the combination therapy, and the next immune mechanism has not been explored. Currently, it is only limited to the illustration of recruited immune cells.

Response: Thank you for pointing this out. In this study, we aimed to investigate the frequency of different immune cells and did not investigate the subsequent mechanisms. We consider this in future studies that are ongoing and we added this suggestion in the discussion part of the manuscript of the article.

3. Since we all know the effectiveness of PD-1 antibody combined with radiotherapy, why do the authors choose CD47 antibody instead of PD-1 antibody? Are there specific groups of people? Or specific tumor subtypes? Is there any cooperation with concurrent clinical research? No experiment on toxicity caused by immunotherapy or combination therapy is described. Even if the program improves the tumor growth rate and survival time, it is not explained whether there are toxic adverse reactions.

Response: We thank you for your attention. In other projects carried out by our research group, we have used the ligand of PD-1 (PDL1) and investigated the efficiency of inhibiting this pathway in combination with radiotherapy [1, 2]. Therefore, we intended to investigate another treatment combination along with radiotherapy. As we mentioned in the introduction of the article, CD47 is overexpressed in a large number of cancers. Also, there is evidence regarding the role of CD47 in T cell exhaustion. Although RT kills tumor cells, it can damage normal cells adjacent to the tumor, limiting RT effectiveness. CD47 Inhibition in combination with RT can protect normal cells against RT while increasing the sensitivity of tumor cells to RT and impairing tumor progression.

So far, most clinical research has been related to the efficacy of a different type of CD47 blocking antibodies and combined treatment of these antibodies with chemotherapy drugs. Also, Anti CD47/PD-1 bifunctional Antibody efficacy is being investigated in clinical research.

About the toxicity caused by this combination therapy, no clinical signs were observed apart from tumor growth. َAlso, No significant weight loss or change in the appearance of the mice was not recorded in any of the animals. We added this in the discussion part of the manuscript of the article.

4. It is recommended to conduct further validation in human xenograft vectors (PDX/mini-PDX/organoids(.

Response: Thank you for suggesting this.

Journal Requirements:

Please review your reference list to ensure that it is complete and correct.

Response: Thank you for pointing this out. We reviewed the reference list.

**References:**

1.	Alinezhad M, Bakhshandeh M, Rostami E, Alimohamadi R, Mosaffa N, Jalali SA (2020) Synergistic effects of anti-PDL-1 with ablative radiation comparing to other regimens with same biological effect dose based on different immunogenic response. PloS one.15(4):e0231507. <https://doi.org/10.1371/journal.pone.0231507>

2.	Ghaffari-Nazari H, Alimohammadi M, Alimohammadi R, Rostami E, Bakhshandeh M, Webster TJ, et al. (2022) Radiation dose and schedule influence the abscopal effect in a bilateral murine CT26 tumor model. International immunopharmacology.108:108737. <https://doi.org/10.1016/j.intimp.2022.108737>
